# Risk factors and prevalence of taeniasis among the Karen people of Tha Song Yang District, Tak Province, Thailand

**DOI:** 10.1051/parasite/2021041

**Published:** 2021-06-18

**Authors:** Teera Kusolsuk, Kittipong Chaisiri, Akkarin Poodeepiyasawad, Surapol Sa-Nguankiat, Nirundorn Homsuwan, Tetsuya Yanagida, Munehiro Okamoto, Dorn Watthanakulpanich, Jitra Waikagul, Paron Dekumyoy, Chalit Komalamisra, Akira Ito

**Affiliations:** 1 Department of Helminthology, Faculty of Tropical Medicine, Mahidol University 420/6 Ratchawithi Road Ratchathewi 10400 Bangkok Thailand; 2 Mahidol Bangkok School of Tropical Medicine, Faculty of Tropical Medicine, Mahidol University 420/6 Ratchawithi Road Ratchathewi 10400 Bangkok Thailand; 3 Laboratory of Veterinary Parasitology, Joint Faculty of Veterinary Medicine 1677-1 Yoshida Yamaguchi City Yamaguchi 753-8511 Japan; 4 Primate Research Institute, Kyoto University Inuyama Aichi 484-8506 Japan; 5 Department of Parasitology, Asahikawa Medical University Midorigaoka-Higashi 2-1-1-1 Asahikawa 078-8510 Hokkaido Japan

**Keywords:** Taeniasis, Kato’s thick smear, PCR, Epidemiological survey

## Abstract

Taeniasis remains a prevalent public health problem in Thailand. National helminthiasis surveys report only the incidence of *Taenia* spp. eggs. The ability to differentiate *Taenia* species using morphological and molecular techniques is vital for epidemiological surveys. This study detected taeniasis carriers and other helminthic infections by Kato’s thick smear technique and identified the *Taenia* species by multiplex PCR. The study subjects were the ethnic Karen people in Tha Song Yang District, Tak Province, Thailand, bordering Myanmar. In total, 983 faecal samples from villagers were examined for helminthiases. Interview-based questionnaires were used to gather information on possible risk factors for infection. The prevalence of helminth infections was 42.7% (420/983), including single (37.3%, 367/983) and mixed infections (5.4%, 53/983). The most common infection (19.23%, 189/983) was *Ascaris lumbricoides*, whereas taeniasis carriers comprised 2.8% (28/983). Multiplex PCR of *Cox1* was used for species identification of *Taenia* tapeworms, eggs, or both in 22 taeniasis carriers. Most of the parasites (20 cases) were *Taenia solium*, with two cases of *Taenia saginata*. *Taenia saginata asiatica* was not found in the villagers examined. The analysis of 314 completed questionnaires showed that a statistically significant (*p* < 0.05) risk of taeniasis was correlated with being male, a history of being allowed to forage during childhood, a history of seeing tapeworm proglottids, and a history of raw or undercooked pork consumption. Health education programmes must seek to reduce and prevent reinfection in these communities.

## Abstract (fr)

La téniase reste un problème de santé publique répandu en Thaïlande. Les enquêtes nationales sur les helminthiases ne rapportent que l’incidence des œufs de *Taenia* spp. La capacité de différencier les espèces de *Taenia* à l’aide de techniques morphologiques et moléculaires est vitale pour les enquêtes épidémiologiques. Cette étude a détecté des porteurs de téniase et d’autres infections helminthiques par la technique de frottis épais de Kato et a identifié les espèces de *Taenia* par PCR multiplex. Les sujets de l’étude étaient les Karens du district de Tha Song Yang, province de Tak, Thaïlande, à la frontière du Myanmar. Au total, 983 échantillons de matières fécales provenant de villageois ont été examinés pour les helminthiases. Des questionnaires basés sur des entretiens ont été utilisés pour recueillir des informations sur les facteurs de risque possibles d’infection. La prévalence des helminthes était de 42,7 % (420/983), dont des infections uniques (37,3 %, 367/983) et mixtes (5,4 %, 53/983). L’infection la plus courante (19,23 %, 189/983) était *Ascaris lumbricoides*, tandis que les porteurs de téniase représentaient 2,8 % (28/983). La PCR multiplexe de *Cox1* a été utilisée pour l’identification des adultes ou des oeufs de *Taenia*, ou des deux, chez 22 porteurs de téniase. La plupart des parasites (20 cas) étaient *Taenia solium*, avec deux cas de *Taenia saginata*. *Taenia saginata asiatica* n’a pas été trouvé chez les villageois examinés. L’analyse de 314 questionnaires a montré qu’un risque statistiquement significatif (*p* < 0,05) de téniase était en corrélation avec le fait d’être un homme, et des antécédents d’avoir été autorisé à ramasser sa nourriture pendant l’enfance, d’avoir vu des proglottis de ténia et de consommation de porc cru ou insuffisamment cuit. Les programmes d’éducation sanitaire doivent chercher à réduire et à prévenir la réinfection dans ces communautés.

## Introduction

Human taeniasis is caused by ingesting undercooked pork carrying the tapeworm *Taenia solium*. It is a significant zoonotic disease because it may cause cysticercosis in the tapeworm carriers, family members, and other close contacts [26]. The World Health Organization (WHO) recognises taeniasis as one of 17 neglected tropical diseases that affect poor people worldwide [7, 32]. *Taenia solium* taeniasis and cysticercosis are endemic in several developing countries where pigs are raised as a food source [6, 30]. Many parts of the world, including Asia, Africa, and Latin America, are endemic areas of these diseases [2, 4, 8, 24]. Humans and pigs acquire cysticercosis by the faecal-oral route by ingesting *T. solium* eggs [5]. After ingestion, the embryos in the eggs are released and cross the intestinal mucosa, from which they are transported by the circulatory system and dispersed throughout the body, mainly producing cysts in the central nervous system and striated muscles [5]. Pigs are infected when reared in areas lacking adequate sanitary infrastructure, where they can feed on human faeces [3]. Although several other parasitic diseases cause mortality, *T. solium* cysticercosis is one of the most lethal parasitic diseases and is the most important foodborne parasite. In terms of health and economic burden, *T. solium* has been ranked as the first, and *Taenia saginata* as the nineteenth foodborne parasite at the global level [31]. In 2015, the WHO Foodborne Disease Burden Epidemiology Reference Group identified *T. solium* as a leading cause of death from foodborne diseases [32]. Prevention of community spread is challenging [2, 6], and control of cysticercosis at the community level is a priority. In Thailand, especially along the Thai–Myanmar border, taeniasis remains a serious medical problem [20]. In 2005, two studies in Nan Province, northern Thailand, showed that 1.9% and 2.4% of people in Nan Province were taeniasis carriers [19, 21]. Patients with cysticercosis are reported only at specialised hospitals in Bangkok, Thailand. In 1997, 25 cases of cysticercosis were reported at Rajvithi Hospital. The highest infection rates were observed in the Northern Province (44%), followed by the central (32%), northeastern (20%), and southern provinces (4%) of Thailand [28]. Prasat Neurological Hospital reported 98 cases, again primarily from the north (27.6%), Bangkok (25.5%), and the central region (25.5%), followed by the northeast (11.2%) and east (10.2%) [10]. This study used faecal examinations for the primary screening of taeniasis carriers and molecular identification of the *Taenia* species to identify the risks associated with taeniasis among the Karen people in Nong Bua, Klur Klor and Tala Okar villages, Tha Song Yang District, Tak Province, Thailand.

## Materials and methods

### Study site and sampling

A cross-sectional study was conducted in three villages of Tha Song Yang District between 2011 and 2013. Tak Province is located in northern Thailand, the province located along the Moei River as a border between Thailand and Myanmar. The target area is a large community of the Karen people and they predominantly work in the forest. Open defecation using outdoor pit latrines is common, and livestock access to these latrine areas is often unrestricted. The practise of non-confinement of pigs is common and slaughter activities are often performed in backyards. The sample size (*N* = 388) was calculated according to the Yamane formula [35] using 5% error and a confidence coefficient of 95%. The population of the Mae U-Su Subdistrict is about 18,000 people (public health data). Nong Bua village is a large Karen community located along the Moei River. Nong Bua is one of ten villages in the subdistrict, classified by landscape into sub-villages (Nong Bua, Klur Klor and Tala Okar). We randomly selected participants, due to the local habit of eating raw pork during special events. The participants had not been examined previously. The sample included both male and female participants aged 15+ years. We distributed 400 questionnaires with faecal containers. We also provided a free medical examination without the questionnaire to all villagers. Recruitment yielded a total pool of 983 faecal samples examined using Kato’s thick smear technique in a field laboratory to detect helminth eggs (particularly *Taenia)*. Faecal containers were distributed to all participants who provided their signed informed consent. Of the 400 sample kits distributed with questionnaires, only 316 were completed; the remaining 84 were incomplete or not returned. This project was approved by the Ethics Committee of the Faculty of Tropical Medicine, Mahidol University No. MUTM 2013-018-04.

### Detection of *Taenia* spp. and other helminth eggs in faeces

A total of 983 faecal samples were examined by Kato’s thick smear technique with special attention to *Taenia* spp. eggs. In 1954, Kato and Miura were the first to introduce the ‘cellophane thick smear technique’ [13], from which the Kato thick smear technique was adapted for control programmes in Japan [12]. This technique is considered the most reliable and practical method for detecting even low-level helminthic infections [12, 14].

The taeniasis carriers were offered deworming treatment. They were asked to evacuate their bowels and eat a non-fibre meal the night before treatment. Early the next morning, four tablets of niclosamide (0.5 g/tablet) were administered, followed by a purgative 2 h later (60 mL saturated magnesium sulphate), and large quantities of drinking water. After treatment, all the evacuated faecal material was collected 4–5 times per individual. The collected materials were repeatedly washed with water until clean, and tapeworm samples were collected from the sediment and processed for further study. The segments of each worm were fixed separately in 95% ethanol for molecular studies [19].

### Molecular identification of *Taenia* spp.

Total DNA was prepared from parasite materials using a DNeasy tissue kit (Qiagen, Hilden, Germany). Cropro-DNA from the faecal samples of tapeworm carriers was extracted using the QIAamp DNA stool Mini kit, which requires at least 0.2 g faeces (Qiagen). Multiplex PCR (mPCR) was performed as described [36] to amplify mitochondrial *Cox1*. The amplified products were electrophoresed on 0.9%–1.0% agarose gels. Reactions were performed in 50 μL volumes to minimise the effect of inhibitors in the copro-DNA samples.

### Assessment for associated risks and statistical analysis

Questionnaires were used to identify the risk factors associated with *Taenia* spp. infection. The standard questionnaire included geographical distribution, risky behaviours such as history of raw pork consumption, open defecation, and free-foraging pigs in the home, and was distributed with faecal containers. Faecal samples were returned to the team and health interviews conducted by volunteers. We worked with local health volunteers who provided Karen language translation for the assessments. Data were analysed using SPSS Windows, version 16.0. Chi-square tests were used to identify the risk factors associated with *Taenia* spp. infection. *P*-values less than 0.05 were considered statistically significant.

### Geographic information system (GIS) mapping of taeniasis carriers

The taeniasis carriers were plotted against the number of pigs raised in the three villages using a GIS recorder.

## Results

Among 983 villagers (588 females and 395 males) who submitted faecal samples, helminthic infection was identified in 41.3% of females and 44.8% of males. The overall prevalence of helminthic infection was 42.7% (420/983), with single infections in 37.3% (367/983) and mixed infections in 5.4% (53/983). The most common infection was *Ascaris lumbricoides* (198/983, 19.2%), followed by hookworms (71/983, 7.2%), *Trichuris trichiura* (62/983, 6.3%), *Taenia* spp. (28/983, 2.8%), and minute intestinal flukes (MIF; 17/983, 1.7%). The mixed infections between *Taenia* spp. eggs and the other helminth eggs were not related among five cases (Table 1A and 1B).

**Table 1:** Prevalence of helminths in males and females among villagers. A and B are single and mixed infection cases, respectively.

Sex	No. faeces examined	Helminthic infection rate	Single infection rate	Single infection (A)									
				Al		Tt		Hw		Tae		MIF	
Male	395	177 (44.8%)	157 (39.7%)	58 (14.7%)		39 (9.9%)		35 (8.9%)		20 (5.1%)		5 (1.3%)	
Female	588	243 (41.3%)	210 (35.7%)	131 (22.3%)		23 (3.9%)		36 (6.1%)		8 (1.4%)		12 (2.0%)	
Total	983	420 (42.7%)	367 (37.3%)	189 (19.2%)		62 (6.3%)		71 (7.2%)		28 (2.8%)		17 (1.7%)	
Sex	No. of faeces examined	Mixed infections	Type of mixed infection (B)										
			Al-Tt	Al-Hw	Tae-Al	Tt-Hw	Tae-Hw	Tt-MIF	Tae-MIF	Tae-Al-Tt	Al-Tt-Hw	Tt-Hw-MIF	Tae-Tt-Hw
Male	395	20 (5.1%)	7 (1.8%)	3 (0.8%)	0	4 (1.0%)	2 (0.5%)	0	1 (0.3%)	0	4 (1.0%)	0	0
Female	588	33 (5.6%)	9 (1.5%)	8 (1.3%)	3 (0.5%)	3 (0.5%)	0	2 (0.3%)	0	1 (0.2%)	3 (0.5%)	2 (0.3%)	1 (0.2%)
Total	983	53 (5.4%)	16 (1.6%)	11 (1.1%)	3 (0.3%)	7 (0.7%)	2 (0.2%)	2 (0.2%)	1 (0.1%)	1 (0.1%)	7 (0.7%)	2 (0.2%)	1 (0.1%)

Al: *Ascaris lumbricoides*; Tt: *Trichuris trichiura*; Hw: hookworm; Tae: *Taenia* spp.; MIF: minute intestinal flukes.

Twenty-eight *Taenia* egg carriers diagnosed by coproparasitology were the main target population for this study, and included single and multiple tapeworm infestations (Table 2A and 2B). The highest prevalence of taeniasis was observed in individuals aged 36–45 years (9/257 villagers), followed by 26–35 years (8/256 villagers), 46–55 years (5/145 villagers), 15–25 years (4/178 villagers), 56–65 years (1/86 villagers), and more than 65 years (1/43 villagers). No taeniasis was identified in villagers aged <15 years (0/18). Among the 28 taeniasis carriers, 9 individuals were selected for species identification (patients 3, 4, 5, 13, 14, 17, 19, 22 and 27). Nineteen carriers consented to deworming, but only 13 received treatment and were purged of proglottids, which were then analysed by mPCR. The remaining patients did not participate because they were working elsewhere (patients 8, 10, 15, 16 and 28) and one was a breastfeeding mother (patient 6). Unfortunately, the residual faecal samples for these individuals were insufficient for molecular analysis and were thus unusable for species identification. The collected scolices and gravid proglottids were examined under a microscope for morphological identification of *Taenia* species. Deworming of 13 taeniasis carriers yielded 6 scolices in 6 carriers, and only gravid segments in the remaining 7. Five of the scolices (patients 1, 2, 12, 20 and 21) had a roughly quadrate form, 1 mm in diameter, with four large, deeply cupped suckers and a rostellum armed with two rows of 22–32 hooklets. From their morphology, they were deemed probable *T. solium*. One scolex (collected from patient 18) had four suckers, with no rostellum or hooklets, and was identified as *T. saginata.* The remaining proglottids could not be identified at the species level. No taeniasis carrier had a previous history of symptoms suggestive of cysticercosis, such as epilepsy/seizure or subcutaneous nodules (Table 3).

**Table 2:** Age distribution and prevalence of helminths in population. A and B are single and mixed infection cases, respectively.

Age group	No. of faeces examined	Helminthic infection rates	Single infection rates	Single infection (A)									Mixed infections (B)
				Al		Tt		Hw		Tae		MIF	
<15	18 (1.8%)	10/18 (55.6%)	9 (50.0%)	7 (38.9%)		2 (11.1%)		0		0		0	1 (5.6%)
15–25	178 (18.1%)	74/178 (41.6%)	68 (38.2%)	40 (22.5%)		11 (6.2%)		9 (5.0%)		4 (2.2%)		1 (0.6%)	6 (3.4%)
26–35	256 (26.0%)	95/256 (37.1%)	81 (31.6%)	37 (14.5%)		17 (6.6%)		14 (5.5%)		8 (3.1%)		4 (1.6%)	14 (5.5%)
36–45	257 (26.1%)	108/257 (42.0%)	98 (38.1%)	48 (18.7%)		13 (5.0%)		28 (10.9%)		9 (3.5%)		4 (1.6%)	10 (3.9%)
46–55	145 (14.8%)	66/145 (44.5%)	53 (36.6%)	28 (19.3%)		8 (5.5%)		7 (4.8%)		5 (3.4%)		5 (3.4%)	13 (9.0%)
56–65	86 (8.7%)	45/86 (52.3%)	40 (46.5%)	24 (27.9%)		6 (7.0%)		10 (11.6%)		1 (1.2%)		0	5 (5.8%)
>65	43 (4.4%)	22/43 (51.2%)	18 (41.9%)	5 (11.6%)		5 (11.6%)		3 (7.0%)		1 (2.3%)		3 (7.0%)	4 (9.3%)
Age group	No. of faeces examined	Mixed infections	Type of mixed infection (B)										
			Al-Tt	Al-Hw	Tae-Al	Tt-Hw	Tae-Hw	Tt-MIF	Tae-MIF	Tae-Al-Tt	Al-Tt-Hw	Tt-Hw-MIF	Tae-Tt-Hw
<15	18 (1.8%)	1 (5.5%)	1 (5.5%)	0	0	0	0	0	0	0	0	0	0
15–25	178 (18.1%)	6 (3.4%)	3 (4.1%)	1 (0.6%)	0	1 (0.6%)	0	0	0	0	1 (0.6%)	0	0
26–35	256 (26.0%)	14 (5.5%)	2 (0.8%)	2 (0.8%)	2 (0.8%)	4 (1.6%)	1 (0.4%)	0	0	1 (0.4%)	2 (0.8%)	0	0
36–45	257 (26.1%)	10 (3.9%)	3 (1.2%)	2 (0.8%)	0	1 (0.4%)	0	0	0	0	3 (1.2%)	0	1 (0.4%)
46–55	145 (14.6%)	13 (9.0%)	5 (3.4%)	3 (2.1%)	1 (0.7%)	1 (0.7%)	0	1 (0.7%)	1 (0.7%)	0	1 (0.7%)	0	0
56–65	86 (8.7%)	5 (5.8%)	1 (1.2%)	3 (3.5%)	0	0	0	0	0	0	1 (1.2%)	0	0
>65	43 (4.4%)	4 (9.3%)	1 (2.3%)	0	0	0	1 (2.3%)	0	0	0	0	2 (4.7%)	0

Al: *Ascaris lumbricoides*; Tt: *Trichuris trichiura*; Hw: hookworm; Tae: *Taenia* spp.; MIF: minute intestinal flukes.

**Table 3:** Profiles of 28 *Taenia* carriers.

No.	Sex	Age	History of eating raw meat		Type of testing	Morphological examination		PCR of adult worm			PCR of faecal eggs		
			Pork	Beef		*T. sol*	*T. sag*	*T. sol*	*T. sag*	*T. sag. asia*	*T. sol*	*T. sag*	*T. sag. asia*
1	M	36	Y	N	DW	Y	N	Y	N	N	NA	NA	NA
2	M	16	Y	N	DW	Y	N	Y	N	N	NA	NA	NA
3	M	36	Y	N	FT	NA	NA	NA	NA	NA	Y	N	N
4	F	28	Y	Y	FT	NA	NA	NA	NA	NA	Y	N	N
5	F	31	Y	N	FT	NA	NA	NA	NA	NA	Y	N	N
6	F	29	Y	N	NA	NA	NA	NA	NA	NA	NA	NA	NA
7	F	28	Y	N	DW	NA	NA	Y	N	N	NA	NA	NA
8	F	31	Y	N	NA	NA	NA	NA	NA	NA	NA	NA	NA
9	M	56	Y	N	DW	NA	NA	Y	N	N	NA	NA	NA
10	M	16	Y	N	NA	NA	NA	NA	NA	NA	NA	NA	NA
11	M	45	Y	N	DW	NA	NA	Y	N	N	NA	NA	NA
12	M	67	Y	N	DW	Y	N	Y	N	N	NA	NA	NA
13	F	25	Y	N	FT	NA	NA	NA	NA	NA	Y	N	N
14	M	36	Y	N	FT	NA	NA	NA	NA	NA	Y	N	N
15	M	51	Y	N	NA	NA	NA	NA	NA	NA	NA	NA	NA
16	F	34	Y	Y	NA	NA	NA	NA	NA	NA	NA	NA	NA
17	M	36	Y	N	FT	NA	NA	NA	NA	NA	Y	N	N
18	M	15	Y	Y	DW	N	Y	N	Y	N	N	N	N
19	F	54	Y	N	FT	NA	NA	NA	NA	NA	Y	N	N
20	M	51	Y	N	DW	Y	N	Y	N	N	NA	NA	NA
21	M	54	Y	N	DW	Y	N	Y	N	N	NA	NA	NA
22	F	36	Y	Y	FT	NA	NA	NA	NA	NA	N	Y	N
23	F	32	Y	N	DW	NA	NA	Y	N	N	NA	NA	NA
24	M	46	Y	Y	DW	NA	NA	Y	N	N	NA	NA	NA
25	M	41	Y	N	DW	NA	NA	Y	N	N	NA	NA	NA
26	M	37	Y	N	DW	NA	NA	Y	N	N	NA	NA	NA
27	M	32	Y	N	FT	NA	NA	NA	NA	NA	Y	N	N
28	M	44	Y	N	NA	NA	NA	NA	NA	NA	NA	NA	NA

M: male; F: female; Y, yes; N, no; NA, not accessible; DW, deworm; FT, faecal test mPCR, *T. sol* = *Taenia solium*; *T. sag* = *Taenia saginata*; *T. sag. asia* = *Taenia saginata asiatica.*

**Figure 1:**
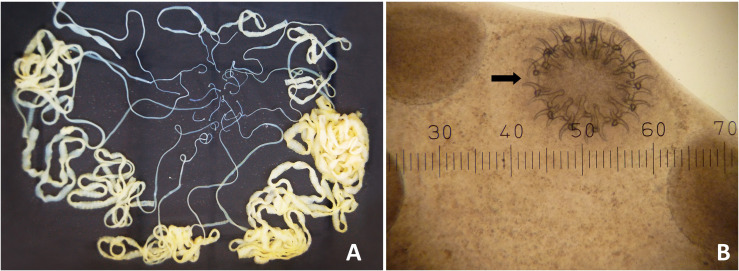
Nineteen *Taenia solium* tapeworms expelled from a 67-year-old Karen male patient (A). Macro-morphology of 19 tapeworms: Round scolices were observed in 19 worms. Morphology of the scolex of an intact worm (40×): The scolex was characterized by two rows of hooklets (the black arrow referred) on the rostellum besides four suckers (B).

Patient 12, a 67 year-old male, had no detectable health problems and only minimal abdominal disturbance. He reported observing tapeworm proglottids in his faeces on a few occasions during the previous two months. He had a 50+ year history of consuming uncooked meat, especially pork and beef. He frequently consumed a traditional raw pork dish called ‘Lahb Moo’ while drinking alcohol with his neighbours. After treatment, 19 scolices and tapeworm chains, each approximately 1 m long, were recovered from his whole faecal samples (Fig. 1A). The scolices were examined under a microscope and identified as *T. solium* (Fig. 1B). Scolices and gravid segments from nine individual worms were gathered and sent to the Department of Parasitology, Asahikawa Medical University, Hokkaido, Japan for molecular identification. Haplotype analysis and mPCR confirmed the samples to be an Asian genotype of *T. solium* (data not shown).

### Questionnaire

Randomly selected participants (*n* = 316) from three villages were surveyed by our group and local health volunteers. The participants included 118 (37.3%) males and 198 (62.7%) females, aged 15–80 years. The number of subjects was highest in the 20–40 age group (149, 47.2%), followed by 41–60 (123, 38.9%). The most common occupation was housewife or housekeeper (118, 37.3%), followed by agricultural labourer in the field or forest, such as maize farmer and hunter (111, 35.1%) (Table 4).

**Table 4:** Demographic data of villagers participating in the questionnaire survey in the three villages studied.

Variables	*N* = 316		
Sex			
Male	118 (37.3%)		
Female	198 (62.7%)		
Variables	Male	Female	Total (*N* = 316)
Age group			
<20 years old	10 (3.2%)	10 (3.2%)	20 (6.3%)
20–40 years old	42 (13.3%)	107 (33.7%)	149 (47.2%)
41–60 years old	58 (18.4%)	65 (20.6%)	123 (38.9%)
>60 years old	8 (2.5%)	16 (5.1%)	24 (7.6%)
Occupation			
Agriculture	55 (17.4%)	56 (17.7%)	111 (35.1%)
Merchant	3 (0.9%)	6 (1.9%)	9 (2.8%)
Student	10 (3.2%)	10 (3.2%)	20 (6.3%)
Labourer	37 (11.7%)	21 (6.6%)	58 (18.4%)
Housewife/Steward	13 (4.1%)	105 (33.2%)	118 (37.3%)

Analysis of possible risk factors for taeniasis showed that males had a 4.0-fold greater risk of taeniasis than females (95% confidence interval [CI] = 1.758–9.239). The villagers who raised pigs under or around their homes were 5.8 times more likely to be infected than those who did not (95% CI = 1.732–19.882). Previous tapeworm infection was a predisposing factor, and villagers who had a history of tapeworm proglottid excretion in their faeces had a 4.5-fold greater chance of infection than villagers with no such history (95% CI = 2.038–10.166). Such recollections were significantly associated with *Taenia* infection (*p* < 0.05). History of consuming raw or undercooked pork increased the risk of infection 5-fold (95% CI = 1.160–21.555). Other factors associated with infection, such as a history of raw beef consumption and mode of defecation, were not associated with taeniasis in this study (Table 5).

**Table 5:** Occurrence of taeniasis in relation to risk factors.

Risk factor (*N* = )	Taeniasis		Odds ratio (95% CI)	*p* value
	Yes	No		
Sex				
Male (118)	19	99	4.030 (1.758–9.239)	0.001*
Female (198)	9	189		
Age				
<30 (55)	6	49	1.330 (0.513–3.452)	0.556
≥30 (261)	22	239		
Raising Pigs				
Yes (194)	25	169	5.868 (1.732–19.882)	0.001*
No (122)	3	119		
Method of raising pigs				
Free (86)	19	67	7.562 (2.892–19.772)	0.001*
Crated/Restrained (166)	6	160		
History of faecal excretion of tapeworm proglottids				
Yes (90)	17	73	4.552 (2.038–10.166)	0.001*
No (226)	11	215		
History of raw pork consumption				
Yes (234)	26	208	5.000 (1.160 – 21.555)	0.022*
No (82)	2	80		
History of raw beef consumption				
Yes (86)	11	75	1.838 (0.823 – 4.101)	0.180
No (228)	17	213		
Method of defecation				
Open defecation (46)	5	41	1.310 (0.471–3.639)	0.604
Toilet (270)	23	247		

### GIS mapping

**Figure 2:**
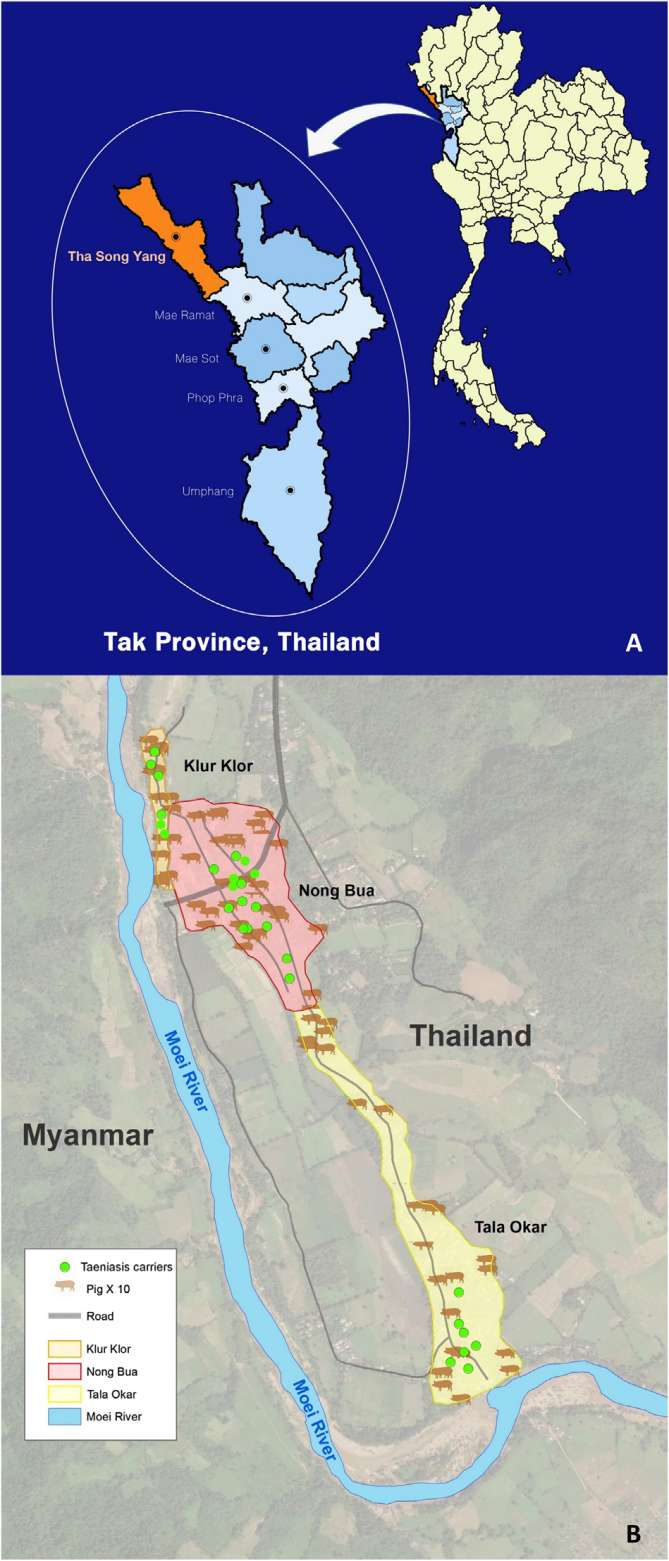
Tha Song Yang District, Tak Province, Thailand, area of the research studied (A). The distribution of 28 taeniasis carriers and number of pigs among three sub-villages (Nong Bua, Klur Klor and Tala Okar), Mae U-Su Sub District, Tha Song Yang District, Tak Province using GIS mapping (B).

GIS technology was used to analyse the distribution of taeniasis carriers and pig-farming activities in the villages. In the study areas, Nong Bua had 84 households and kept about 280 pigs, Klur Klor had about 63 households and about 147 pigs, and Tala Okar had about 71 households with about 213 pigs. The villagers raise pigs for food and for sale to their neighbours, and for breeding. A total of 28 taeniasis-positive carriers were recorded using GPS. The highest prevalence of taeniasis was in Nong Bua, with 15 taeniasis carriers (green dots), followed by six cases in Tala Okar and seven cases in Klur Klor (Figs. 1B, 2A, 2B).

### Molecular identification of *Taenia* spp. from eggs/proglottids

Species identification by mPCR was performed in 66 samples (53 faecal samples and 13 gravid segments). The faecal samples were selected after they had been examined for helminth eggs with Kato’s thick smear technique and divided into three categories: (1) 9 were positive for *Taenia* spp. eggs; (2) 19 were positive for eggs of other helminths; and (3) 25 were negative for any helminth. The negative samples were randomly selected and as controls to verify primer specificity. The results showed that all nine *Taenia*-positive faecal samples generated a PCR product diagnosis for taeniasis, 8 were identified as *T. solium* and one was confirmed as *T. saginata*. The *Taenia* scolices and long proglottids were collected from 13 taeniasis carriers and preserved in 95% ethyl alcohol as well. mPCR showed that 12 were *T. solium,* one case was identified as *T. saginata*, and there were no results for *T. saginata asiatica*. Faecal samples positive for other helminths or negative for any helminth did not generate a PCR product diagnosis for taeniasis.

## Discussion

The helminth infection rate among the villagers was high (420/983; 42.7%), with a taeniasis infection rate of 2.8% (28/983). In 2014, a national survey of helminthiasis in Thailand reported infection rates of 18.1% for helminthiases and 0.7% for *Taenia* spp. [33]. Therefore, our results for total helminthiases and taeniasis among Karen people were extremely high compared with the general population of Thailand. In 2015, McCleery et al. reported a high prevalence (2.9%) of taeniasis among refugees living on the Thai–Myanmar border using copro-parasitological examination [20]. In 2015, Kaewpitoon and colleagues conducted a cross-sectional survey of intestinal helminthiases in rural communities of Nakhon Rachasima Province, northeastern Thailand, and reported a 6.2% overall prevalence of helminthic infections, the most common being hookworm (4.3%), whereas the incidence of taeniasis was 0.48% [11]. They concluded that age and agricultural occupation were risk factors for helminthic infections, consistent with our findings. In this study, taeniasis carriers were mapped with GPS to determine their distribution patterns in the Sub-Villages relative to the distribution of pigs raised in the same area (Fig. 2B). In these villages, pigs are raised for meat and can roam freely to forage. The survey results showed that 26 of 28 *Taenia* carriers had a history of raw pork consumption. Some people still use open latrines around the village. These are important factors in the persistence of taeniasis in this population.

Ascariasis was the most common helminth infection (189/983, 19.2%), followed by hookworm infection (71/983, 7.2%), trichuriasis (47/983, 4.8%), and taeniasis (28/983, 2.8%). Similar results were reported in another village in Tha Song Yang District, Tak Province, where the incidence of ascariasis was 47.8% (32/67), followed by trichuriasis (7/67, 10.5%), hookworm (4/67, 5.9%), and taeniasis (1/67, 1.5%) [18]. The prevalence of *Ascaris* was the highest among individuals <15 years old (7/18, 38.9%). This high prevalence of ascariasis can be attributed to lifestyle habits of drinking untreated water, not washing hands before meals, and defecating in outside latrines near their houses. Poor hygiene is the main cause of persistent helminthiases, especially soil-transmitted helminths. The implementation of a health education programme to improve hygiene was recommended as critical to reduce and prevent reinfection. The *Taenia* infections in the three villages studied here represent a difficult challenge. The villagers prefer to allow pigs to forage around their houses and villages. Some households do not have a toilet, but inhabitants defecate outside, which facilitates the transmission of *Taenia* eggs to the pigs and other villagers. The local villagers’ lifestyle means that when they have special celebratory events, such as weddings or New Year, they kill infected pigs and eat raw or undercooked pork. These practices are risk factors for helminthic infections, especially taeniasis.

The study areas are located along the Moei River on the Thai–Myanmar border, and some villagers, especially male workers, frequently cross the river to work in the fields or vegetable gardens or to hunt in the forest. These occupations are common among the hill tribe people. More than half (194/316, 61.4%) of the study population raises pigs beneath their houses, for food, to sell the pigs or piglets to other households, or for breeding. A history of pig rearing was an important, statistically significant risk factor for taeniasis (*p* < 0.05). Many villagers allow their pigs to forage around their houses and villages because it is less expensive than keeping the pigs corralled. Some villagers defecate outside, risking the spread of tapeworm proglottids in the environment, which are then taken up as food by the free-range pigs and subsequently transmitted to humans. In India, Sankhyan et al. reported that of 182 patients with epilepsy, only 22 (12%) believed that a worm was responsible for their seizures, whereas 94 (52%) were aware of the link between worms and epilepsy [25]. Despite this knowledge, 150 (82%) patients were unsure of the name or nature of the worm. In fact, 89 (49%) believed that the worm was acquired by eating cabbages rather than by consuming pork or poor personal hygiene. Only 14 (8%) patients had ever consumed pork. In this study, taeniasis carriers were present in this community and the infection persisted according to the “complete triangle” concept of the parasite: in the “host” (the villagers, who enjoy eating raw pork), the “agent” (*T. solium* proglottids passed in faeces from infected humans), and the “environment” (pigs become infected with metacestodes after ingesting *T. solium* eggs from proglottids in the faeces of infected humans). In fact, 74.1% (234/316) of the villagers reported consuming raw pork, maintaining the high prevalence of taeniasis carriers in the community.

When we analysed the characteristics of the taeniasis carriers, the prevalence was highest in the middle-aged groups, ranging from 36–45 years old (9/28, 32.1%) to 26–35 years old (8/28, 28.6%). The taeniasis carriers were predominantly engaged in agriculture, working in corn fields, on vegetable crops or hunting animals in the forest. They may have become infected by cooking at their work places or returning from work with meat. Drinking alcohol and consuming local dishes as a group every evening are common and typically include uncooked or raw pork dishes, such as “Lahb” or “Loo”. Among 28 taeniasis patients, most (26/28) declared a history of consuming raw or undercooked pork.

The implementation of health programmes is essential to alter local cultural and habitual practices, for the sustainable improvement of the villagers’ environment. In 2015, Wandra and colleagues successfully applied this approach to the prevention and control of taeniasis in Indonesia. They concluded that a treatment programme for human taeniasis carriers, with pig vaccination, is necessary for the successful control of taeniasis and neurocysticercosis in endemic areas [29]. A history of faecal proglottid tapeworm is an important factor in screening for taeniasis. In this study, a questionnaire revealed that 88 of the interviewed villagers had a history of passing proglottids in their faeces. Nevertheless, *Taenia* eggs were confirmed in only 28 villagers with microscopic faecal examinations. Some villagers were not interviewed and some were unsure about seeing proglottids in their faeces. Possible reasons include (1) low sensitivity of Kato’s thick smear test; small number of eggs; (2) mismatch in the timing of faecal sampling and the release of eggs from gravid segments; and (3) detached gravid segments in faeces on regular defecation and mature segments initially developing to gravid segments on the sampling time. The infection might also have been underestimated because inadequate information was available at the interviews.

The prevalence of *Taenia* egg detection in this study was associated with sex, with a significantly higher (4.0 times) risk in males than in females (*p* < 0.05). Anantaphruti et al. also reported that in more than 400 taeniasis carriers in Thailand, the male:female ratio was 1.9:1 [1]. A higher risk of taeniasis in males was also reported in Vietnam, where it was noted that males commonly consume undercooked beef, pork, or pork visceral organs while drinking alcohol as part of traditional cultural practices [22]. In our study, the lifestyles of adult men, in terms of their social activities and occupations, may put them at risk of taeniasis. In terms of the sanitary conditions of the communities studied, about 14.6% (46/316) of the villagers interviewed defecate in pit latrines, the bush or shedding areas. These facilitate the transmission of taeniasis between humans and pigs in this area. Most of the *Taenia* tapeworm species collected in this study were identified morphologically as *T. solium* by their scolices. Only few samples with ill-defined scolices were ultimately identified with mPCR. Two samples were *T. saginata*, but not *T. saginata asiatica*. In Thailand, there has been a single report of *T. saginata asiatica* in a villager who lived in a refugee camp near the Thai–Myanmar border in Kanchanaburi Province [1].

In this study, there was no taeniasis reported in children (<15 years old), but the sample was too small to conclude that children are at no risk of infection. In other studies, the highest risk in children (<15 years old) was reported in the Philippines [34], China [15, 23, 36], and Congo [17]. Also, the life span of *T. solium* is estimated <3–5 years [6, 9, 16]. Thus, further studies focussing on school children <15 years old should be conducted. Risk information should include how old children are when they start eating pork, since countries where *T. solium* taeniasis infection has been identified in children, pork, especially minced pork, is served as baby food [27].

## Conclusion

The infection rate of helminthiases in the study area was 42.7%, including 37.3% single infections and 5.4% mixed infections. The commonest helminth was *Ascaris lumbricoides* (19.2%), followed by hookworm (7.2%), *Trichuris trichiura* (6.3%), *Taenia* spp. (2.8%), and minute intestinal flukes (1.7%). mPCR of *Cox1* identified 13 *Taenia*-proglottid-positive samples. Nine faecal samples positive for *Taenia* eggs were identified as *T. solium* and only two samples were *T. saginata*. The risk factors for taeniasis were male sex, a history of pig rearing, and allowing pigs to forage freely, a history of faecal excreta of tapeworm proglottids, and a history of raw or undercooked pork consumption were statistically significant (*p* < 0.05). The villagers maintain the cultural practices of eating raw pork, using open latrines, and raising free-range pigs. The vicious cycle of taeniasis among the population will continue until such practices are moderated.

The implementation of an intensive control programme, including proper treatment, health education, changing the practice of raising free-range pigs, avoiding the consumption of raw pork or beef, and improvements in the quality of life, such as a clean water supply and latrines, is required in this community. A follow-up is recommended to re-evaluate the taeniasis carriers in the study area.

## Conflict of interest

The authors declare no conflict of interest related to this work.

## References

[1] Anantaphruti MT, Yamasaki H, Nakao M, Waikagul J, Watthanakulpanich D, Nuamtanong S, Maipanich W, Pubampen S, Sanguankiat S, Muennoo C, Nakaya K, Sato MO, Sako Y, Okamoto M, Ito A. 2007. Sympatric occurrence of *Taenia solium*, *T. saginata*, and *T. asiatica*, Thailand. Emerging Infectious Diseases, 13(9), 1413–1416. 18252126 10.3201/eid1309.061148 PMC2857269

[2] Bruno E, Bartoloni A, Zammarchi L, Strohmeyer M, Bartalesi F, Bustos JA, Santivañez S, García HH, Nicoletti A, Group CPS. 2013. Epilepsy and neurocysticercosis in Latin America: a systematic review and meta-analysis. PLoS Neglected Tropical Diseases, 7(10), e2480. 24205415 10.1371/journal.pntd.0002480 PMC3814340

[3] Carpio A. 2002. Neurocysticercosis: an update. Lancet Infectious Diseases, 2(12), 751–762. 12467692 10.1016/s1473-3099(02)00454-1

[4] Fabiani S, Bruschi F. 2013. Neurocysticercosis in Europe: still a public health concern not only for imported cases. Acta Tropica, 128(1), 18–26. 23871891 10.1016/j.actatropica.2013.06.020

[5] García HH, Gilman RH, Gonzalez AE, Verastegui M, Rodriguez S, Gavidia C, Tsang VC, Falcon N, Lescano AG, Moulton LH. 2003. Hyperendemic human and porcine *Taenia solium* infection in Peru. American Journal of Tropical Medicine and Hygiene, 68(3), 268–275. 12685628

[6] García HH, Gonzalez AE, Evans CA, Gilman RH, Peru CWGi. 2003. *Taenia solium* cysticercosis. Lancet, 362(9383), 547–556. 12932389 10.1016/S0140-6736(03)14117-7 PMC3103219

[7] Hotez PJ, Molyneux DH, Fenwick A, Ottesen E, Sachs SE, Sachs JD. 2006. Incorporating a rapid-impact package for neglected tropical diseases with programs for HIV/AIDS, tuberculosis, and malaria. PLoS Medicine, 3(5), e102. 16435908 10.1371/journal.pmed.0030102 PMC1351920

[8] Ito A, Nakao M, Wandra T, Suroso T, Okamoto M, Yamasaki H, Sako Y, Nakaya K. 2005. Taeniasis and cysticercosis in Asia and the Pacific: present state of knowledge and perspectives. Southeast Asian Journal of Tropical Medicine and Public Health, 36, 123. 16438196

[9] Ito A, Saito M, Donadeu M, Lightowlers MW. 2020. Kozen Yoshino’s experimental infections with *Taenia solium* tapeworms: An experiment never to be repeated. Acta Tropica, 205, 105378. 32057776 10.1016/j.actatropica.2020.105378

[10] Jitsukon N. 1989. Neurocysticercosis at Prasat Neurological Hospital. Bulletin of the Department of Medical Services, 14, 289–300.

[11] Kaewpitoon SJ, Loyd RA, Kaewpitoon N. 2015. A cross-sectional survey of intestinal helminthiases in rural communities of Nakhon Ratchasima province, Thailand. Journal of the Medical Association of Thailand, 98(suppl 4), 27–32. 26201131

[12] Kato K. 1960. A correct application of the thick-smear technique with cellophane paper cover. A pamphlet. Japanese Journal of Medical Science and Biology, 19, 58–64.

[13] Kato K, Miura M. 1954. Comparative examinations Jap J Parasitol 3: 35 (Japanese text). Apud Martin LK, Beaver PC, 1968. Evaluation of Kato thick-smear technique for quantitative diagnosis of helminth infections. American Journal of Tropical Medicine and Hygiene, 17, 382–391. 10.4269/ajtmh.1968.17.382 5690644

[14] Komiya Y, Kobayashi A. 1966. Evaluation of Kato’s thick smear technic with a cellophane cover for helminth eggs in feces. Japanese Journal of Medical Science and Biology, 19(1), 59–64. 5296934 10.7883/yoken1952.19.59

[15] Li T, Chen X, Wang H, Openshaw JJ, Zhong B, Felt SA, Ito A, Luby SP. 2019. High prevalence of taeniasis and *Taenia solium* cysticercosis in children in western Sichuan, China. Acta Tropica, 199, 105133. 31415736 10.1016/j.actatropica.2019.105133

[16] Lightowlers MW. 1999. Eradication of *Taenia solium* cysticercosis: a role for vaccination of pigs. International Journal for Parasitology, 29(6), 811–817. 10480718 10.1016/s0020-7519(99)00051-x

[17] Madinga J, Kanobana K, Lukanu P, Abatih E, Baloji S, Linsuke S, Praet N, Kapinga S, Polman K, Lutumba P. 2017. Geospatial and age-related patterns of *Taenia solium* taeniasis in the rural health zone of Kimpese, Democratic Republic of Congo. Acta Tropica, 165, 100–109. 26996821 10.1016/j.actatropica.2016.03.013 PMC5178865

[18] Maipanich W, Dekumyoy P, Sa-Nguankiat S, Pubampen S, Poodeepiyasawat A, Watthanakulpanich D. 2014. Houseflies with helminthic objects, good indicator of an unsanitary environment. Proceedings of the Joint International Tropical Medicine Meeting, 3, 40–46.

[19] Maipanich W, Sato M, Pubampen S, Sanguankiat S, Kusolsuk T, Thaenkham U, Waikagul J. 2011. Abnormal *Taenia saginata* tapeworms in Thailand. Southeast Asian Journal of Tropical Medicine and Public Health, 42(5), 1065. 22299430

[20] McCleery EJ, Patchanee P, Pongsopawijit P, Chailangkarn S, Tiwananthagorn S, Jongchansittoe P, Dantrakool A, Morakote N, Phyu H, Wilkins PP. 2015. Taeniasis among refugees living on Thailand-Myanmar border, 2012. Emerging Infectious Diseases, 21(10), 1824. 26401787 10.3201/eid2110.141657 PMC4593425

[21] Muennoo C, Waikagul J, Maipanich W, Sanguankiat S, Pubampen S, Watthanakulpanich D, Nuamtanong S, Yoonuan T. 2005. Liver fluke and minute intestinal fluke infection in Sa Kaeo and Nan provinces, Thailand. Journal of Tropical Medicine and Parasitology, 28(1), 16–21.

[22] Ng-Nguyen D, Stevenson MA, Breen K, Van Phan T, Nguyen V-AT, Van Vo T, Traub RJ. 2018. The epidemiology of *Taenia* spp. infection and *Taenia solium* cysticerci exposure in humans in the Central Highlands of Vietnam. BMC Infectious Diseases, 18(1), 1–9. 30348095 10.1186/s12879-018-3434-9 PMC6198533

[23] Openshaw JJ, Medina A, Felt SA, Li T, Huan Z, Rozelle S, Luby SP. 2018. Prevalence and risk factors for *Taenia solium* cysticercosis in school-aged children: A school based study in western Sichuan, People’s Republic of China. PLoS Neglected Tropical Diseases, 12(5), e0006465. 29738570 10.1371/journal.pntd.0006465 PMC5959190

[24] Praet N, Speybroeck N, Manzanedo R, Berkvens D, Nforninwe DN, Zoli A, Quet F, Preux P-M, Carabin H, Geerts S. 2009. The disease burden of *Taenia solium* cysticercosis in Cameroon. PLoS Neglected Tropical Diseases, 3(3), e406. 19333365 10.1371/journal.pntd.0000406 PMC2656639

[25] Sankhyan P, Gupta S, Singh G. 2015. Knowledge about, attitudes towards, practices regarding *Taenia solium* cysticercosis among people attending an epilepsy clinic in India. International Journal of Epilepsy, 2(1), 6–10.

[26] Schantz PM, Cruz M, Sarti E, Pawlowski ZJ. 1993. Potential eradicability of taeniasis and cysticercosis. Bulletin of the Pan American Health Organization, 27(4), 397–403. 8312963

[27] Swastika K, Wandra T, Dharmawan NS, Sudarmaja IM, Saragih JM, Diarthini LPE, Ariwati L, Damayanti PAA, Laksemi DAAS, Kapti N, Sutisna P, Yanagida T, Ito A. 2017. Taeniasis caused by *Taenia saginata* in Gianyar town and *Taenia solium* in Karangasem villages of Bali, Indonesia, 2011–2016: How to detect tapeworm carriers, anamnesis or microscopy? Acta Tropica, 174, 19–23. 28634145 10.1016/j.actatropica.2017.06.013

[28] Techathuvanan S. 1997. Cysticercosis: 5-year review at Rajavithi Hospital. Journal of the Rajavithi Hospital, 8, 33–41.

[29] Wandra T, Swastika K, Dharmawan NS, Purba IE, Sudarmaja IM, Yoshida T, Sako Y, Okamoto M, Diarthini NLPE, Laksemi DAAS, Yanagida T, Nakao M, Ito A. 2015. The present situation and towards the prevention and control of neurocysticercosis on the tropical island, Bali. Indonesia. Parasites & Vectors, 8(1), 1–11. 10.1186/s13071-015-0755-z PMC4356148 25881045

[30] White AC Jr. 2000. Neurocysticercosis: updates on epidemiology, pathogenesis, diagnosis, and management. Annual Review of Medicine, 51(1), 187–206. 10.1146/annurev.med.51.1.187 10774460

[31] WHO. 2014. Multicriteria-based ranking for risk management of food-borne parasites: report of a Joint FAO: FAO, World Health Organization pp.

[32] WHO. 2014. Taeniasis/Cysticercosis. 2015 [cited 2020 15 October]; Available from: http://www.who.int/mediacentre/factsheets/fs376/en/.

[33] Wongsaroj T, Nithikathkul C, Rojkitikul W, Nakaia W, Royal L, Rammasut P. 2014. National survey of helminthiasis in Thailand. Asian Biomedicine, 8(6), 779–783.

[34] Xu J-M, Acosta LP, Hou M, Manalo DL, Jiz M, Jarilla B, Pablo AO, Ovleda RM, Langdon G, McGarvey ST, Kurtis JD, Friedman JF, Wu H-W. 2010. Seroprevalence of cysticercosis in children and young adults living in a helminth endemic community in Leyte, the Philippines. Journal of Tropical Medicine, 2010, 603174. 20368794 10.1155/2010/603174 PMC2846682

[35] Yamane T. 1967. Statistics: An introductory analysis, 2nd Ed. New York: Harper and Row.

[36] Yamasaki H, Allan JC, Sato MO, Nakao M, Sako Y, Nakaya K, Qiu D, Mamuti W, Craig PS, Ito A. 2004. DNA differential diagnosis of taeniasis and cysticercosis by multiplex PCR. Journal of Clinical Microbiology, 42(2), 548–553. 14766815 10.1128/JCM.42.2.548-553.2004 PMC344500

